# Musical Sonification of Ligand-Engineered CsPbBr_3_ Quantum Dots with Enhanced Stability

**DOI:** 10.3390/nano16201290

**Published:** 2026-10-11

**Authors:** Cheng Sun, Yajuan Wang, Zhiyang Xu, Jianing Wang, Wenyan Liu

**Affiliations:** 1 Department of Music, Beihua University, Jilin City 132013, China; 2 Department of Materials Science and Engineering, Beihua University, Jilin City 132013, China; 3 Department of Basic Disciplines, Nanchang Jiaotong University, Nanchang 330100, China; 4 Changchun Institute of Optics, Fine Mechanics and Physics, Chinese Academy of Sciences, Changchun 130033, China

**Keywords:** CsPbBr_3_ quantum dots, ligand modification, stability, data sonification

## Abstract

CsPbBr_3_ quantum dots (QDs) have been widely applied in lighting, detection, display, and many other fields. However, due to their ionic crystal nature, further improvement in stability is still required for practical applications. To address this issue, this study employed a ligand engineering strategy to synthesize tributylphosphine (TBP)-modified CsPbBr_3_ QDs in a Zn^2+^-containing reaction system. Experimental results demonstrate that the modified QDs exhibit superior optical properties, as well as significantly enhanced thermal stability and resistance to UV photodegradation. The introduction of Zn^2+^ into the reaction system leads to a more uniform size distribution, improved monodispersity, and enhanced crystallinity of CsPbBr_3_ QDs. More importantly, the photostability of CsPbBr_3_ QDs under varied temperatures, cyclic heating–cooling processes, and prolonged UV irradiation is notably improved after Zn^2+^ incorporation. Furthermore, the TBP-modified CsPbBr_3_ QDs synthesized with Zn^2+^ assistance exhibit further enhanced photostability under all the aforementioned aging conditions. Additionally, we introduce a data-sonification method that translates the temporal evolution of photoluminescence spectra into audible audio signals using an oboe timbre, enabling auditory visualization of the aging process. This work not only provides an effective strategy for stabilizing perovskite QDs but also opens new possibilities for the application of luminescent materials in interactive displays.

## 1. Introduction

Since the first report of all-inorganic perovskite quantum dots CsPbX_3_ QDs (X = Cl, Br, I) by Kovalenko and co-workers in 2015, colloidal CsPbX_3_ QDs have attracted extensive attention due to their excellent optical properties [1,2,3]. Lead halide perovskite QDs, featuring high optical gain, tunable emission across the entire visible spectrum, narrow emission linewidths, and high photoluminescence quantum yields (PLQY), have emerged as a class of novel optical materials with great application potential in the optoelectronic field [4,5]. These materials are highly promising for lighting and display applications and have significantly advanced the development of these fields [6].

However, CsPbX_3_ QDs are susceptible to degradation upon exposure to oxygen, ultraviolet (UV) light, and moisture in air. In particular, polar organic solvents, water, and UV irradiation accelerate the decomposition of CsPbX_3_ QDs, accompanied by PL quenching [7,8]. The relatively poor stability resulting from these factors has become a critical bottleneck constraining their further development [9].

Numerous factors affect the thermal stability of perovskite quantum dots, among which the most critical one is the non-uniform size distribution, which leads to increased defect density within the crystals. During continuous heating, internal stress inhomogeneity develops within the perovskite QD lattice, causing lattice distortion that ultimately leads to the collapse of the perovskite structure. Strategies to address this issue can be broadly classified into two categories: (i) “encapsulation” of perovskite quantum dot materials to shield them from external environmental factors, and (ii) ligand modification of quantum dots to passivate surface defects, improve particle size distribution, and enhance dispersity [10].

Encapsulation strategies embed QDs in inert matrices to shield them from external environmental factors. For example, CsPbBr_3_ QDs embedded in borosilicate glass retained 52.1% of their initial intensity at 373 K and about 97.0% of their luminescence after 10 days of water immersion [11]. CsPbBr_3_ QDs encapsulated in silica aerogel maintained 99% of their initial PL intensity after 10 heating–cooling cycles between 25 and 150 °C [12]. Metal–organic framework encapsulation has shown similar effects; CsPbBr_3_@γ-CD-MOFs achieved a PLQY of 73% along with excellent thermal and photochemical stability [13]. However, encapsulation strategies often introduce additional synthesis steps and may affect the optical properties or device-integration compatibility of QDs.

Ligand modification is another widely studied strategy. Organophosphine ligands such as trioctylphosphine (TOP), TBP, and diphenylphosphine (DPP) are widely used for surface passivation of II-VI, IV-VI, and I-III-VI QDs because the lone pair electrons on the phosphorus atom can form strong coordination with Pb^2+^. In perovskite systems, TOP has been shown to significantly enhance the multiple stabilities of perovskite QDs against long-term storage, temperature, and UV irradiation [14]. Zhang et al. reported a ligand-engineering strategy using TOP combined with lead stearate, where the resulting CsPbBr_3_ QDs showed a 116% increase in PL intensity at 100 °C and a complete failure time of 1900 h in a humid–thermal environment [15]. In addition, a dual-ligand strategy (e.g., DDAB combined with hexylphosphonic acid) achieved a PLQY of 93.7% and retained about 57% of the initial intensity after 450 min of UV irradiation [16]. Fluoride ion passivation and sulfonic acid ligand modification also showed significant effects on UV stability and thermal cycling stability, respectively [17,18]. TBP as a ligand has also been reported for perovskite QDs; TBP-capped perovskite nanocrystals (PeNCs) exhibited enhanced long-term colloidal stability and photostability, as well as higher tolerance to polar solvents [19]. However, systematic studies on the synergistic effect of TBP and Zn^2+^ on the stability of CsPbBr_3_ QDs remain limited.

It is well established that organophosphine ligands such as TBP are commonly employed as capping ligands for II-VI, IV-VI, and I-III-VI quantum dots, including CdSe, PbS, and CuInSe_2_ QDs [20,21,22,23], due to their ability to coordinate with the QD surface and form a dense passivation layer. Building upon this, the present study adopts a ligand-assisted reprecipitation (LARP) method to synthesize TBP-modified CsPbBr_3_ QDs. Meanwhile, Zn^2+^ is introduced into the reaction system to synthesize both Zn^2+^-driven CsPbBr_3_ QDs and TBP-modified CsPbBr_3_ QDs, enabling a systematic investigation of the synergistic effects of ligand modification and Zn^2+^ incorporation on the optical properties and stability of QDs.

The role of Zn^2+^ in perovskite QDs has been widely reported, but mainly focused on lattice doping. For example, incorporating Zn^2+^ into the Pb sites of CsPbBr_3_ NCs via hot injection can boost the PLQY to 91.3% [24]. Zn-doped CsPbBr_3_ PeNCs coated with ZnO showed an increase in PLQY from 50% to over 84%, and with NiO coating it reached 90% [25]. Zn^2+^ ion doping has also been demonstrated to improve the stability of NCs under dilution and illumination, and to reduce blinking on sub-millisecond timescales. However, these works all position Zn^2+^ as a lattice dopant, which is fundamentally different from the role of Zn^2+^ as a reaction mediator in this work.

Furthermore, this study introduces an innovative cross-modal data presentation approach—data sonification. Conventional spectral data presentation methods, such as charts and plots, often fail to intuitively convey the dynamic evolution of materials during aging processes. Sonification technology addresses this limitation by mapping data into audible audio signals, offering a new dimension for perceiving material degradation. Specifically, we map the peak position, intensity, and full width at half maximum (FWHM) of PL spectra at different aging stages (under UV irradiation and thermal cycling) to pitch, volume, and timbre, respectively, thereby transforming the degradation process of quantum dot stability into a time-evolving “auditory fingerprint.” This sonification approach not only provides an intuitive and convenient supplementary tool for real-time monitoring of material aging but also opens new possibilities for the application of luminescent materials in emerging fields such as interactive displays and human–machine interfaces.

In summary, this work focuses on CsPbBr_3_ QDs, employing a synergistic strategy of TBP ligand modification and Zn^2+^ incorporation to enhance their optical stability. The PL evolution of the materials under UV irradiation and thermal cycling aging conditions is systematically characterized, and the application potential of sonification for auditory visualization of material aging processes is further explored.

## 2. Materials and Methods

### 2.1. Materials

Lead bromide (PbBr_2_, 99.99%, Aladdin, Shanghai, China), cesium bromide (CsBr, 99.999%, Aladdin), zinc bromide (ZnBr_2_, 99.9%, Aladdin, Shanghai, China), oleic Acid (OA, 90%, Aladdin, Shanghai, China), oleylamine (OAm, 80–90%, Aladdin, Shanghai, China), N, N-Dimethylformamide (DMF, >99.9%, Aladdin, Shanghai, China), TBP (95%, Aladdin, Shanghai, China), anhydrous ethanol (≥99.7%, DAMAO, Tianjin, China), hexane (97%, DAMAO, Tianjin, China), molecular sieve (3Å, pellets, 3–5 nm, Aladdin, Shanghai, China), 1-octadecene (ODE, ≥90%, Aladdin, Shanghai, China. All the reagents were dehydrated with molecular sieve.

### 2.2. Synthesis

Four types of QDs were synthesized in this work, namely CsPbBr_3_, CsPbBr_3_-TBP, Zn/CsPbBr_3_ and Zn/CsPbBr_3_-TBP. All syntheses were carried out using the ligand-assisted reprecipitation method at 30 °C under ambient air without inert gas protection. The precursor solutions for the three types of QDs were all prepared using 5 mL of DMF as solvent, with the addition of 0.5 mL of OA and 0.25 mL of OAm, and stirred for about 4 h until complete dissolution. The differences among them were as follows: for CsPbBr_3_ QDs, 0.043 g (0.20 mmol) of CsBr and 0.073 g (0.20 mmol) of PbBr_2_ were used without TBP; for CsPbBr_3_-TBP QDs, 0.043 g of CsBr and 0.073 g of PbBr_2_ were used with an extra 0.5 mL of TBP; for Zn/CsPbBr_3_ QDs, the PbBr_2_ amount was reduced to 0.063 g (0.17 mmol) and 0.012 g (0.05 mmol) of ZnBr_2_ was added, also without TBP; for Zn/CsPbBr_3_-TBP QDs, the same Zn-containing formulation was used with an extra 0.5 mL of TBP. After preparing all the precursor solutions, the same purification procedure was applied: 10 mL of n-hexane, 1 mL of precursor solution, and 10 mL of anhydrous ethanol were placed in a 50 mL centrifuge tube, centrifuged at 6000 rpm for 6 min, the supernatant was discarded, and the precipitate was re-dispersed in n-hexane to obtain the corresponding QD samples.

### 2.3. Characterization

The PL spectra and UV irradiation stability measurements were performed on QD samples dispersed in n-hexane. The thermal stability and cyclic heating–cooling tests were performed on QD samples dispersed in ODE. For the optical measurements, the samples were quantitatively diluted to have identical absorbance at the excitation wavelength (405 nm). The temperature range of thermal stability and cyclic heating–cooling tests was 30–100 °C. One complete heating–cooling cycle consisted of heating from 30 °C to 100 °C, holding at 100 °C, and cooling back to 30 °C, taking approximately 2 h in total. All four samples were heated and cooled simultaneously under identical conditions. The UV irradiation experiments were performed using a 365 nm lamp (power: 8 W). The quartz cuvette containing the QD dispersion in n-hexane was placed at a distance of 20 cm from the lamp and irradiated from the side in a dark room. The instrument is not equipped with an internal light-intensity meter, so the absolute incident light intensity (mW/cm^2^) was not calibrated. However, all samples were irradiated under identical distance, geometry, and dark-room conditions to ensure comparability.

The x-ray diffraction (XRD) pattern was obtained using a Bruker D8 ADVANCE diffractometer (Bruker AXS, Karlsruhe, Germany) with Cu Kα radiation (λ = 1.5418 Å), operating at 40 kV and 40 mA, with a scan range of 5–90° (2θ), and a scan speed of 10°/min. The Fourier transform infrared (FTIR) spectrum was measured using Nicolet iS50 FT-IR spectrometer (Thermo Fisher Scientific, Waltham, MA, USA). Transmission electron microscopy (TEM) images were recorded by transmission electron microscopy (JEM F200, JEOL Ltd., Akishima, Japan). Ultraviolet absorption spectrum (UV-vis) was carried out by UV-vis spectrophotometer (UV-2700, Bruker, Karlsruhe, Germany). The PL spectrum was measured using a PL spectrophotometer (Omni PL bh, Zolix, Beijing, China) with an excitation source of 405 nm laser.

## 3. Results

**Figure 1:**
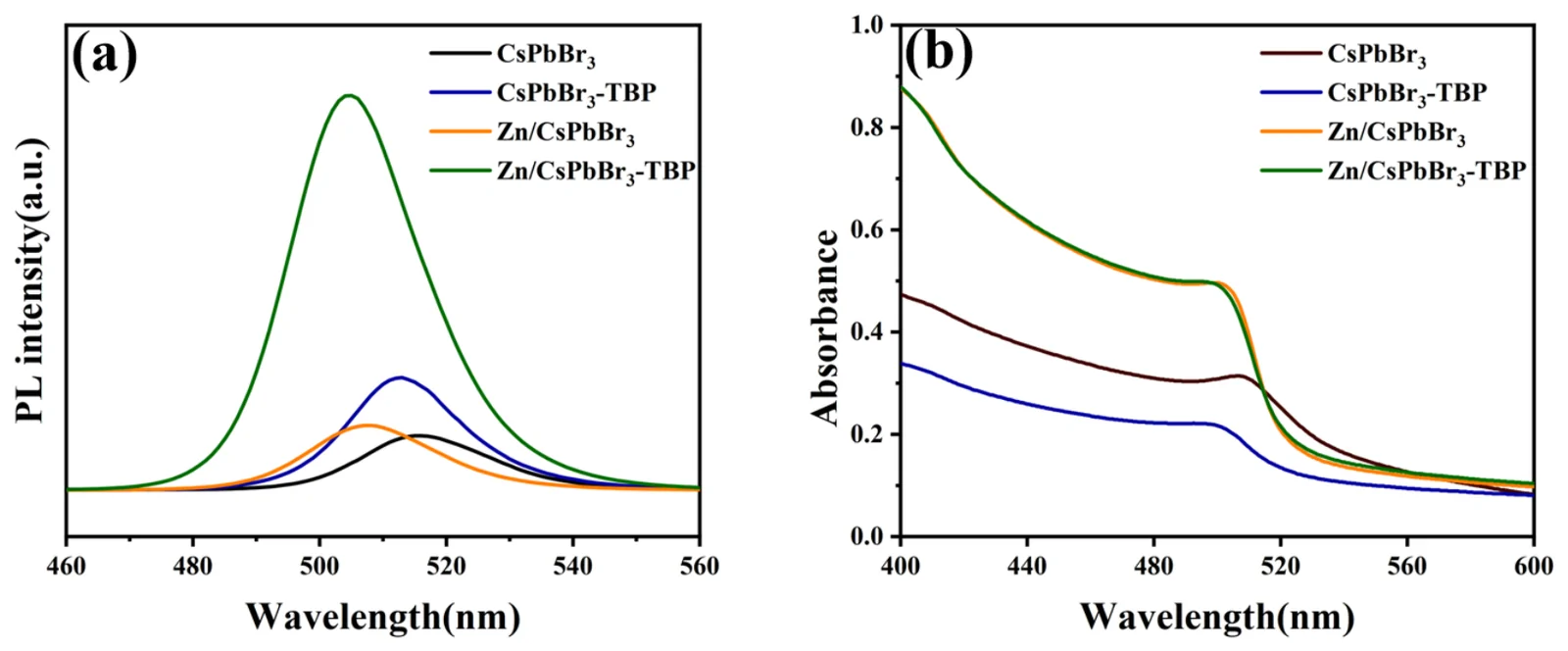
(**a**) PL spectra and (**b**) UV-vis absorption spectra of CsPbBr_3_, CsPbBr_3_-TBP, Zn/CsPbBr_3_ and Zn/CsPbBr_3_-TBP QDs.

The photoluminescence (PL) and UV-vis absorption spectra of CsPbBr_3_, CsPbBr_3_-TBP, Zn/CsPbBr_3_, and Zn/CsPbBr_3_-TBP QDs were measured, as shown in Figure 1. Both TBP modification alone and Zn introduction alone substantially improved the luminescence performance of the QDs, with a more pronounced effect for Zn incorporation. Nevertheless, the best enhancement was achieved when TBP and Zn were introduced simultaneously. Compared with the pristine CsPbBr_3_ QDs, the other three QDs all exhibited blue-shifted PL and absorption spectra, and the blue shift was larger upon Zn introduction. These results suggest a synergistic enhancement between Zn incorporation and TBP modification, which further improves the luminescence performance and stability of the QDs.

**Figure 2:**
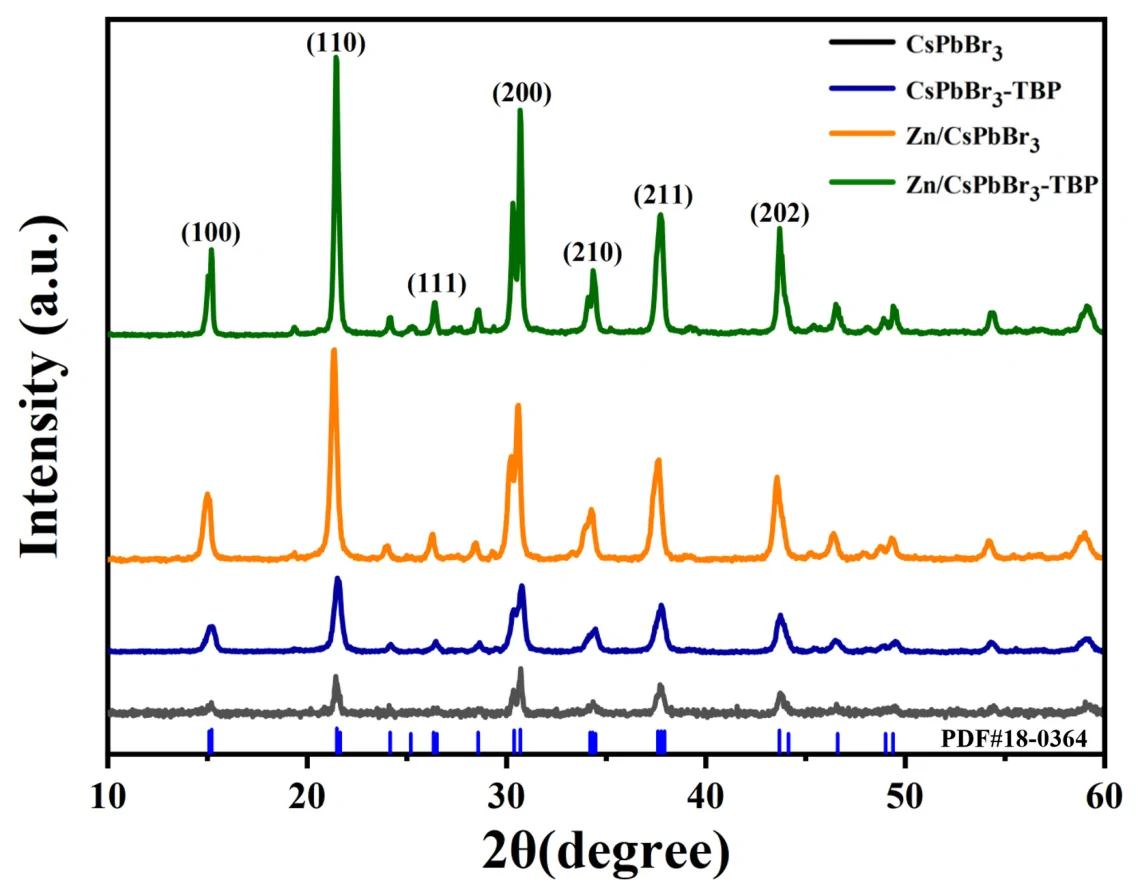
XRD patterns of CsPbBr_3_, CsPbBr_3_-TBP, Zn/CsPbBr_3_ and Zn/CsPbBr_3_-TBP QDs. The intensity of the CsPbBr_3_ pattern (black curve) multiplied by a factor of 2 for clarity.

As shown in Figure 2, all samples exhibit characteristic diffraction peaks at identical 2θ positions (e.g., 15.1°, 21.5°, 30.6°), which are well indexed to the cubic CsPbBr_3_ phase (PDF#18-0364). This confirms that the introduction of Zn^2+^ and TBP does not alter the fundamental crystal structure. For clarity, the intensity of the pristine CsPbBr_3_ pattern (black curve) in Figure 2 was multiplied by a factor of 2 solely to make its weak diffraction peaks visible; this scaling does not alter any peak positions or the original raw data. Notably, compared to the pristine CsPbBr_3_, the modified samples display much sharper and stronger diffraction peaks, indicating a significant improvement in crystallinity. The weak diffraction peaks at higher angles (e.g., 43.7°, 46.5°) are clearly resolved in the modified samples. In the pristine sample, these peaks are also present but exhibit extremely low intensity and poor signal-to-noise ratio, making them barely distinguishable from the background noise due to its lower crystallinity. Notably, the diffraction peaks of Zn/CsPbBr_3_ and Zn/CsPbBr_3_-TBP QDs are significantly enhanced and sharpened, indicating that the crystallinity of both TBP-modified and unmodified CsPbBr_3_ QDs is markedly improved under the synergistic effect of Zn^2+^. These results further demonstrate that Zn^2+^ incorporation plays a positive role in enhancing the performance of CsPbBr_3_ QDs.

**Figure 3:**
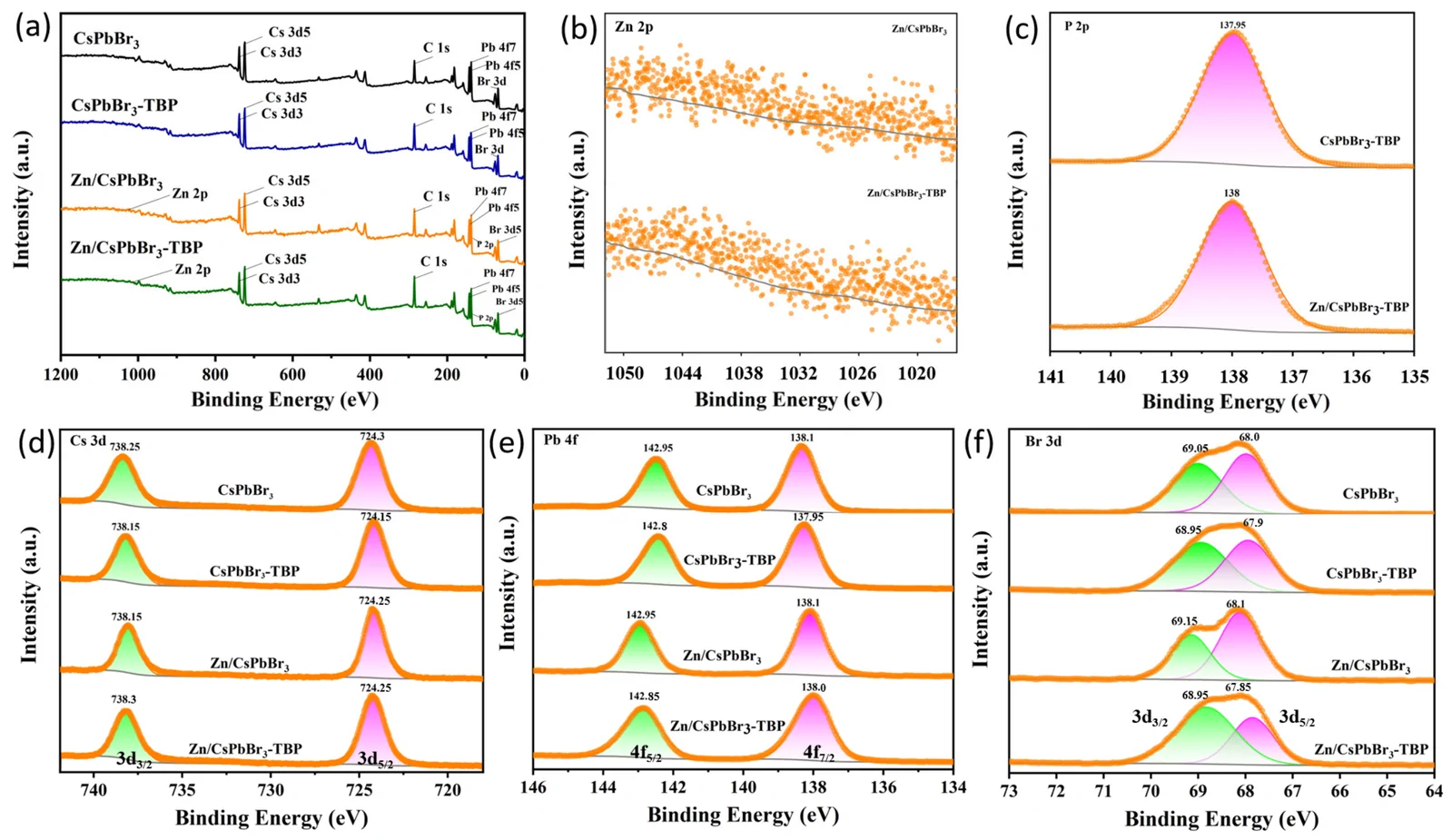
(**a**) Full spectrum of Zn/CsPbBr_3_ and Zn/CsPbBr_3_-TBP QDs, and high-resolution XPS spectra of (**b**) Zn 2p (The circles represent the experimental data.), (**c**) P 2p, (**d**) Cs 3d, (**e**) Pb 4f, (**f**) Br 3d.

To investigate the surface properties of TBP-modified and unmodified CsPbBr_3_ QDs after the introduction of Zn^2+^, X-ray photoelectron spectroscopy (XPS) measurements were performed on the QD samples. The XPS survey spectra shown in Figure 3a confirm the presence of Cs, Pb, and Br elements, as well as Zn and P in the corresponding samples. Compared with the pristine CsPbBr_3_ QDs, the Br 3d signals shift toward lower binding energy upon Zn^2+^ incorporation, indicating that the chemical environment of Br is altered. Furthermore, as revealed in Figure 3d–f, after Zn^2+^ introduction, the Cs 3d_3_/_2_ signal of TBP-modified CsPbBr_3_ QDs shifts to higher binding energy relative to the unmodified counterpart, whereas the Pb 4f and Br 3d signals both shift to lower binding energies. This observation suggests that, under the promoting effect of Zn^2+^, the chemical environment of TBP-modified CsPbBr_3_ QDs is further modified, accompanied by an enhanced interaction between the TBP ligands and the QDs. As shown in Figure 3b, no Zn 2p signal is detected, indicating that the introduced Zn^2+^ does not incorporate into the crystal lattice of CsPbBr_3_ QDs. In contrast, the P 2p signal in Figure 3c confirms the successful coordination of TBP ligands on the QD surface. The XRD and XPS results indicate no significant Zn lattice doping. Zn^2+^ acts as a reaction mediator or surface coordination regulator in the reaction system. It enhances the coordination dynamics and precursor exchange in the reaction medium, thereby modulating the nucleation and growth kinetics and promoting more complete precursor conversion. This ultimately reduces the structural disorder and surface defect/trap density in the final QDs, leading to improved PL performance. In addition, the appearance of the P 2p characteristic peak confirms the successful modification of TBP ligands.

**Figure 4:**
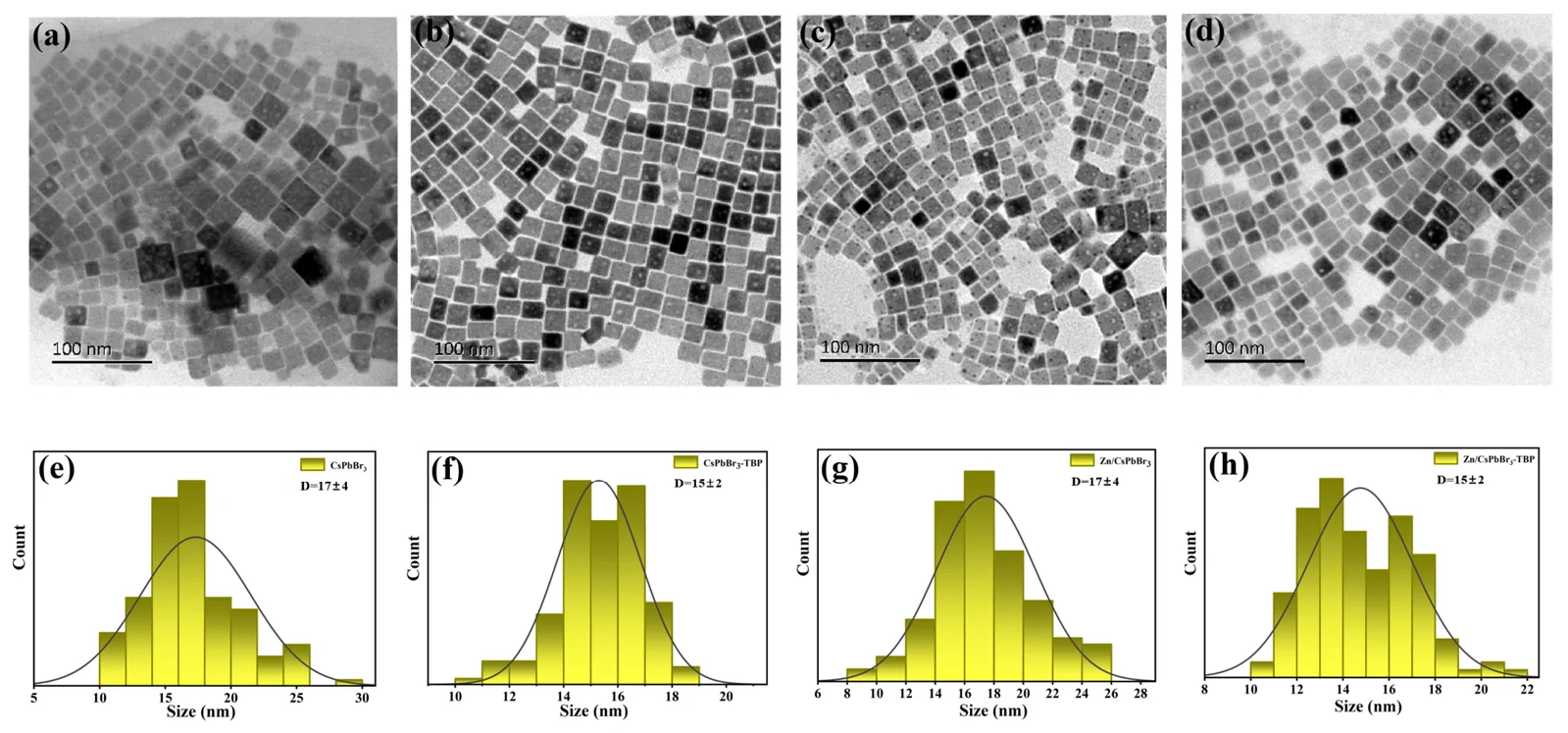
(**a**–**d**) TEM images and (**e**–**h**) grain size distribution of CsPbBr_3_, CsPbBr_3_-TBP, Zn/CsPbBr_3_ and Zn/CsPbBr_3_-TBP QDs.

As shown in Figure 4, the four samples of CsPbBr_3_, CsPbBr_3_-TBP, Zn/CsPbBr_3_ and Zn/CsPbBr_3_-TBP QDs exhibit a cubic perovskite structure with good monodispersity. Compared with the TEM image of pristine CsPbBr_3_ QDs, the Zn-containing QDs show a more uniform size distribution and improved monodispersity after Zn^2+^ introduction. In addition, “black spots” arising from uncoordinated Pb are observed in the TEM image of Zn/CsPbBr_3_ QDs, whereas such black spots are scarcely present in that of Zn/CsPbBr_3_-TBP QDs. This observation indicates that the introduction of TBP ligands effectively removes these black spots, which also implies a high PL intensity. The size distribution histograms shown in Figure 4e–h are very close, and their confidence intervals overlap, so the average sizes of the two samples are statistically indistinguishable.

To investigate the photostability of TBP-modified and unmodified CsPbBr_3_ QDs under the synergistic effect of Zn^2+^, their PL spectra were measured under various conditions, including different temperatures, cyclic heating–cooling processes, and prolonged UV irradiation.

**Figure 5:**
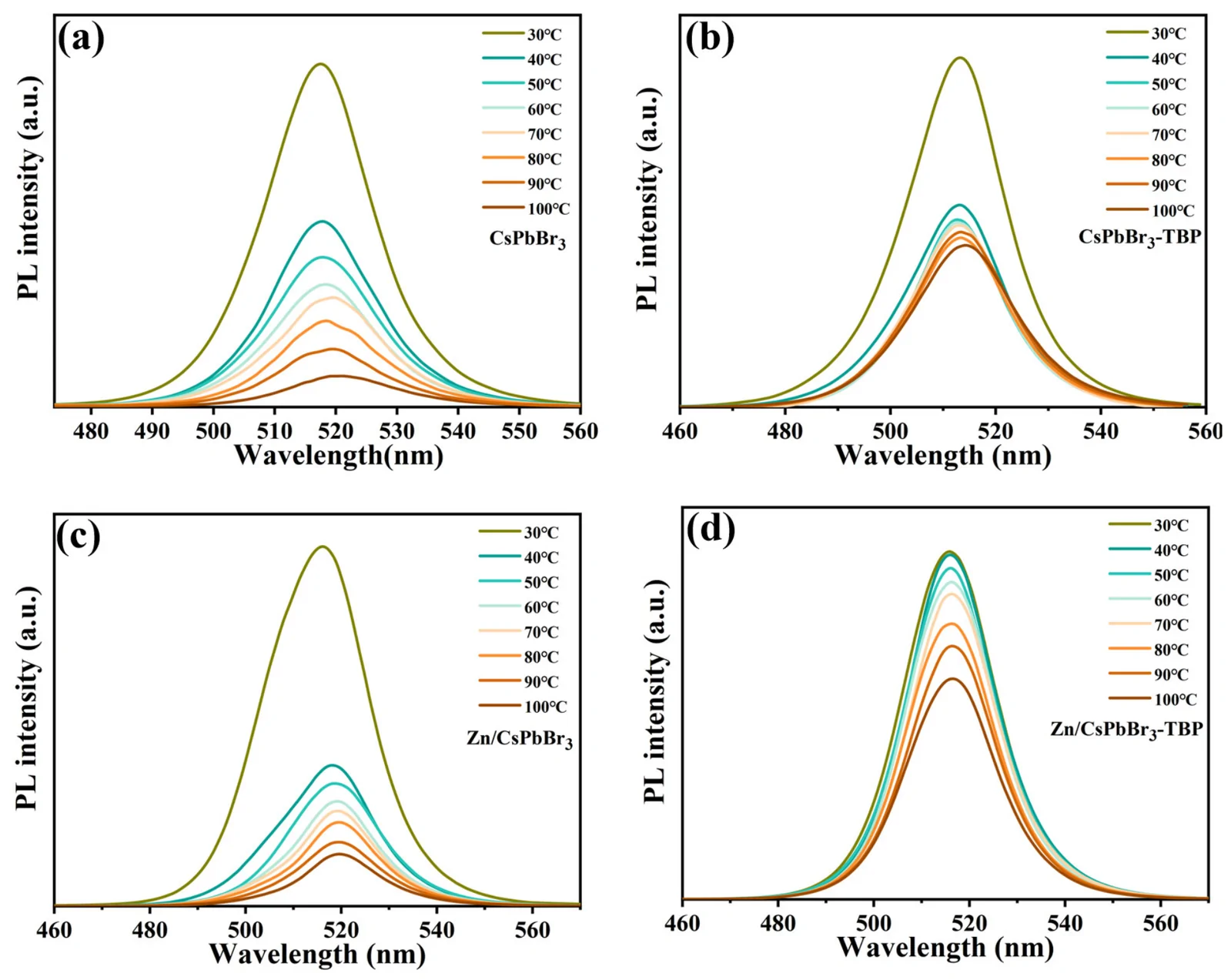
Temperature-dependent PL spectra of (**a**) CsPbBr_3_, (**b**) CsPbBr_3_-TBP, (**c**) Zn/CsPbBr_3_ QDs and (**d**) Zn/CsPbBr_3_-TBP QDs.

The PL spectra of CsPbBr_3_, CsPbBr_3_-TBP, Zn/CsPbBr_3_ and Zn/CsPbBr_3_-TBP QDs at various temperatures were first measured, as shown in Figure 5. With a gradual increase in temperature, the PL intensity of Zn/CsPbBr_3_ QDs decreased rapidly. When heated to 40 °C, the PL intensity dropped to 40% of its initial value, and upon further heating to 100 °C, it decreased to approximately 15% of the original intensity. In contrast, pristine CsPbBr_3_ QDs retained only about 8% of their initial PL intensity at 100 °C. This comparison indicates that the introduction of Zn^2+^ enhances the photostability of CsPbBr_3_ QDs under varying temperatures to a certain extent. In addition, the PL intensity of Zn/CsPbBr_3_-TBP QDs declined more gradually with increasing temperature. At 40 °C, the PL intensity remained at 98% of its initial value, and even at 100 °C, it still retained 63% of its original intensity. This demonstrates that, under the promoting effect of Zn^2+^, the TBP-modified CsPbBr_3_ QDs exhibit excellent thermal stability.

**Figure 6:**
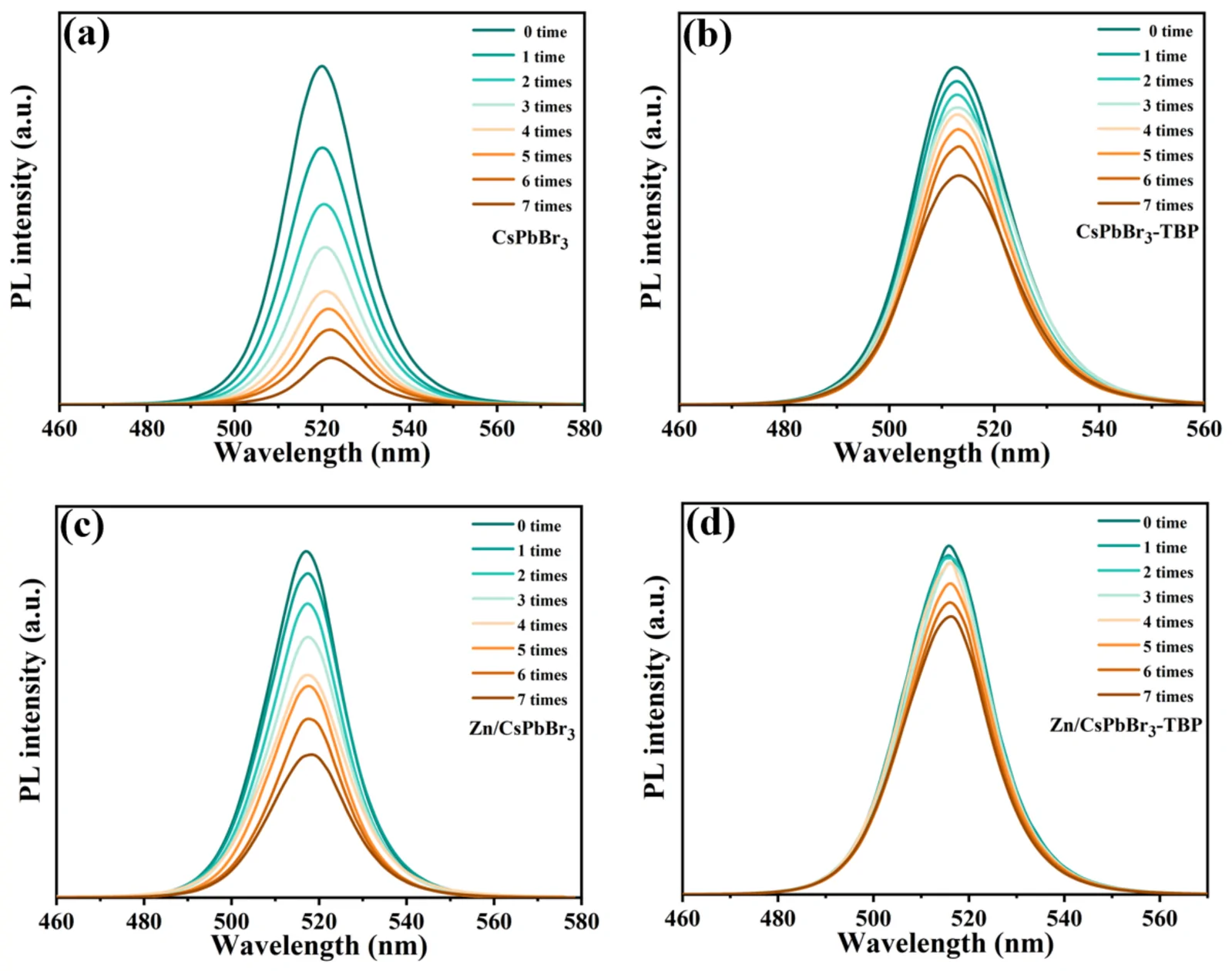
PL spectra of (**a**) CsPbBr_3_, (**b**) CsPbBr_3_-TBP, (**c**) Zn/CsPbBr_3_ QDs and (**d**) Zn/CsPbBr_3_-TBP QDs after cyclic heating to 100 °C and cooling to 30 °C.

Subsequently, the PL spectra of CsPbBr_3_, CsPbBr_3_-TBP, Zn/CsPbBr_3_ and Zn/CsPbBr_3_-TBP QDs were recorded during cyclic heating–cooling processes, as shown in Figure 6. After seven heating–cooling cycles, the PL intensity of Zn/CsPbBr_3_ QDs decreased to 41% of its initial value. By comparison, the PL intensity of CsPbBr_3_ QDs without Zn^2+^ incorporation dropped to only 13% of its original intensity after the same number of cycles. This further confirms that the introduction of Zn^2+^ improves the thermal stability of CsPbBr_3_ QDs. On the other hand, the PL intensity of Zn/CsPbBr_3_-TBP QDs decreased slowly with increasing cycle number, retaining 79% of its initial intensity after seven cycles. This is likely attributed to the passivation layer formed by TBP modification under the promoting effect of Zn^2+^, which shields the Zn/CsPbBr_3_ QDs from temperature-induced degradation.

**Figure 7:**
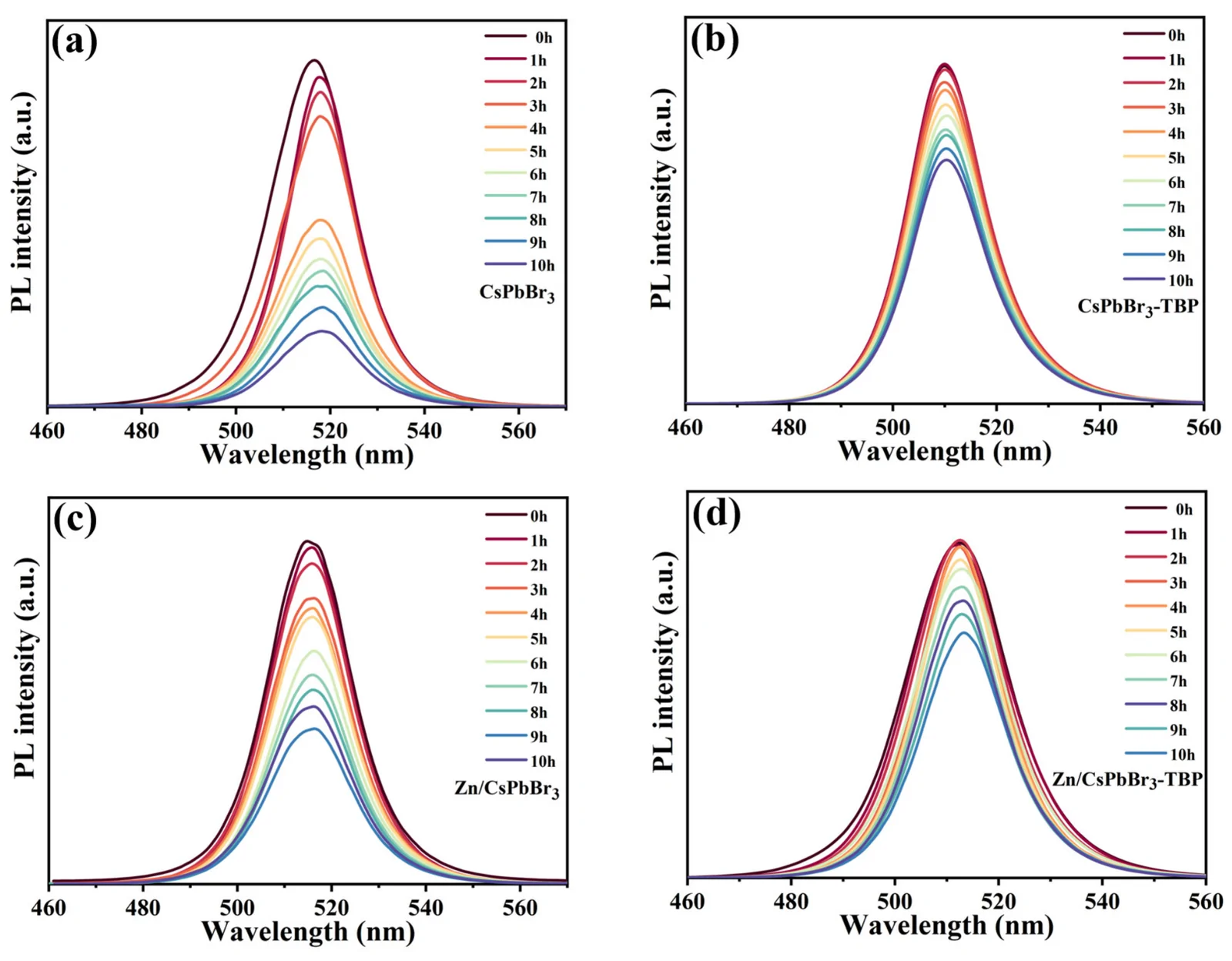
PL spectra of (**a**) CsPbBr_3_, (**b**) CsPbBr_3_-TBP, (**c**) Zn/CsPbBr_3_ QDs and (**d**) Zn/CsPbBr_3_-TBP QDs at 365 nm under ultraviolet irradiation for 10 h.

**Figure 8:**
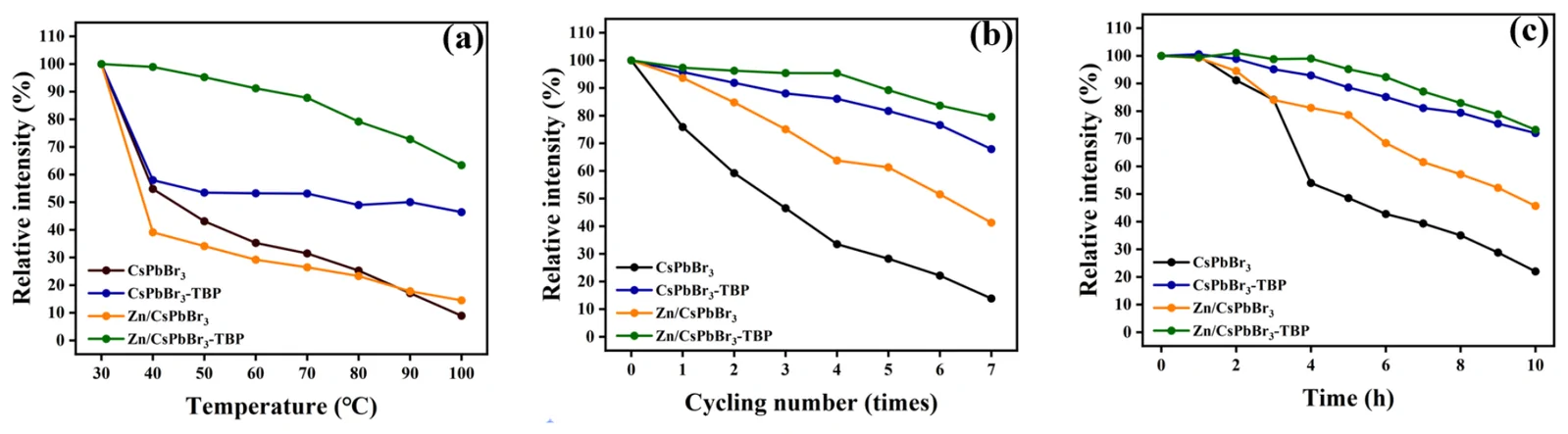
Dot plot of the relative PL intensity changes of CsPbBr_3_, CsPbBr_3_-TBP, Zn/CsPbBr_3_ QDs and Zn/CsPbBr_3_-TBP QDs under (**a**) different temperatures, (**b**) different cycles and (**c**) different irradiation times.

To investigate the photostability of TBP-modified and unmodified CsPbBr_3_ QDs under prolonged UV irradiation after Zn^2+^ introduction, their PL spectra were recorded at hourly intervals. As shown in Figure 7, after 10 h of UV irradiation, the PL intensity of Zn/CsPbBr_3_ QDs decreased to 46% of its initial value, whereas that of pristine CsPbBr_3_ QDs dropped to only 21%. This indicates that the introduction of Zn^2+^ significantly enhances the photostability of CsPbBr_3_ QDs under UV irradiation. In contrast, the PL intensity of Zn/CsPbBr_3_-TBP QDs decreased to only 73% of its initial value after the same irradiation duration, demonstrating that the TBP-modified CsPbBr_3_ QDs exhibit enhanced resistance to UV photodegradation under the promoting effect of Zn^2+^. In addition, after 10 h of UV illumination, the Zn/CsPbBr_3_-TBP QDs remained well dispersed in solution, displaying bright green emission. In contrast, green precipitates were observed at the bottom of the cuvette containing the unmodified QDs, which has been attributed in the literature to the removal of surface-bound ligands upon photon absorption [26]. The dot-line plots in Figure 8, showing the relative PL intensity changes of CsPbBr_3_, CsPbBr_3_-TBP, Zn/CsPbBr_3_ and Zn/CsPbBr_3_-TBP QDs under different temperatures, cycle numbers, and irradiation times, are presented to more intuitively demonstrate that the photostability of TBP-modified CsPbBr_3_ QDs is significantly enhanced under the promoting effect of Zn^2+^ upon addition to the reaction system.

**Figure 9:**
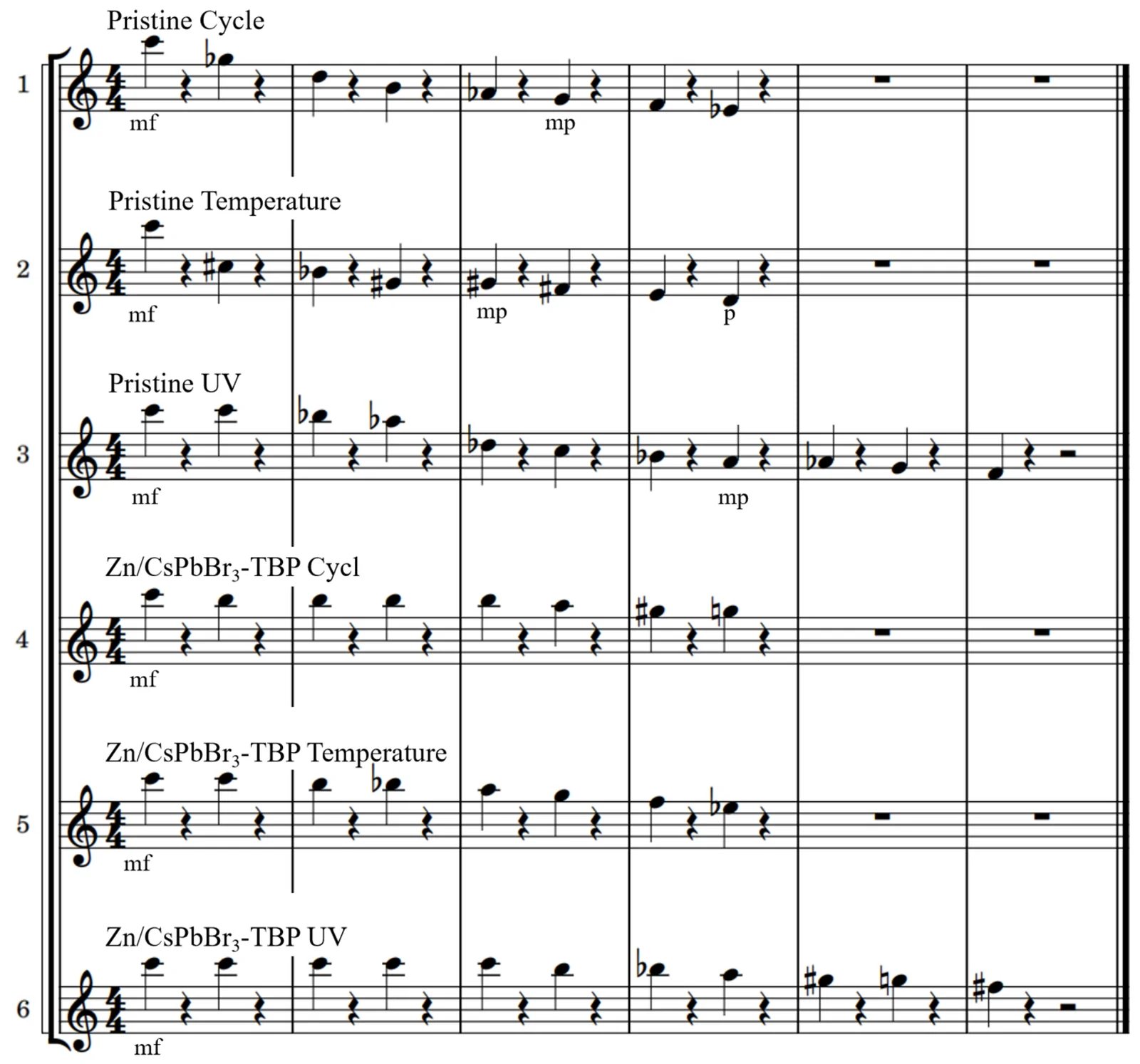
Complete oboe scores generated from sonified PL data of pristine CsPbBr_3_ (top) and Zn/CsPbBr_3_-TBP QDs (bottom) under three aging conditions (cyclic heating–cooling, temperature-dependent, and UV aging, left to right). The dynamic markings in the scores are mf (mezzo-forte, moderately loud), mp (mezzo-piano, moderately soft), and p (piano, soft). The corresponding audio recordings are provided in the Supplementary Materials (Audios S1–S7).

Figure 9 presents the complete oboe scores. The oboe was selected for the sonification because its distinctive and expressive timbre, together with its clear pitch articulation, allows the physicochemical variations of CsPbBr_3_ QDs to be effectively translated into auditory parameters. Its rich harmonic content and penetrating yet warm tone make subtle changes in the data, such as stability-related features, easier to perceive and distinguish. As shown in Figure 9, the top row corresponds to pristine CsPbBr_3_ QDs, and the bottom row to Zn/CsPbBr_3_-TBP QDs. The three columns represent, from left to right, cyclic heating–cooling, temperature-dependent, and UV aging conditions. The sonification was performed using a custom Python script based on the midiutil library. All MIDI files were programmatically generated using Python 3.11.9 and the midiutil 1.2.1 library, after which they were imported into MuseScore Studio 4.6.5 to generate the scores and render them with an oboe timbre. The relative PL intensity (normalized to 100% at the initial state for each sample) was linearly mapped to MIDI note numbers: 100% → C6 (MIDI note 84), 0% → C4 (MIDI note 60), spanning 24 semitones. The mapping formula is MIDI note = 60 + (I/100) × 24, where I is the relative PL intensity in percent. No empirical calibration was involved; the mapping is purely deterministic and data-driven. The resulting MIDI files were imported into MuseScore to generate the oboe scores. The pitch sequence is fully reproducible from the data and was not constrained to a particular musical scale. For completeness, the PL peak position was mapped to dynamic level (MIDI velocity), with larger peak shifts corresponding to softer dynamics. This mapping amplifies the most prominent spectral variation—the dramatic intensity decay—into a clearly audible melodic contour, while the subtle peak shifts are rendered as nuanced dynamic changes.

As shown in Figure 9, a striking visual and auditory contrast is observed between the top and bottom rows. For pristine CsPbBr_3_ QDs (top row), all three fragments exhibit a pronounced downward melodic trajectory, accompanied by a gradual decrease in dynamics, reflecting the substantial loss of PL intensity and the progressive peak shift during aging. Taking the temperature-dependent fragment (Pristine_Temperature) as an example, the pitch starts at C6 (corresponding to 100% initial intensity) and descends to C#4 (corresponding to only 8.9% remaining intensity)—a drop of 19 semitones. This substantial downward melodic contour vividly conveys the rapid decay of emission under thermal stress. The UV aging fragment (Pristine_UV) shows a similar but more gradual descent from C6 to F#4 over 14 semitones, reflecting the slower yet continuous deterioration under prolonged irradiation. The dynamic markings in these fragments progressively soften from mf to p, consistent with the gradual peak shifts observed spectroscopically.

By contrast, the three fragments for Zn/CsPbBr_3_-TBP QDs (bottom row) display a markedly different character, with only a gentle downward pitch trajectory and stable dynamic levels throughout the aging process. For the TBP-modified samples, the pitch remains within a narrow range—from C6 to F5-G#4 (5–7 semitones), reflecting the minimal loss of PL intensity (63–79% retention). The dynamics remain relatively constant (mf to mp), consistent with the suppressed peak shifts observed for these samples. This striking contrast in melodic contour directly visualizes the enhanced photostability achieved through the synergistic effect of Zn^2+^ incorporation and TBP modification: the pristine samples “fall” as they degrade, while the TBP-modified samples “stand firm”.

Beyond its role in materials characterization, the sonification approach presented here carries a dual significance. On one hand, it offers a novel tool for the auditory visualization of material degradation, making complex spectral evolution directly perceptible through the timbral changes in the oboe, and providing an intuitive supplement to conventional monitoring methods. On the other hand, the conversion of scientific data into musical parameters opens up fresh possibilities for artistic creation. We anticipate that this interdisciplinary approach could be extended to the study of other luminescent materials, while also inspiring new forms of data-driven composition and interactive audio-visual display technologies.

## 4. Conclusions

In this paper, we propose a ligand-engineering strategy combined with Zn^2+^ incorporation to synthesize TBP-modified CsPbBr_3_ QDs with substantially improved stability. The modified QDs exhibit not only enhanced optical properties but also remarkable resistance to thermal stress and UV irradiation, outperforming the unmodified counterparts under harsh conditions. Importantly, the introduction of Zn^2+^ plays a critical role in refining the size distribution and crystallinity, which contributes to the overall photostability enhancement. Furthermore, we develop a data-sonification technique that converts the PL spectra into audio signals, offering an intuitive approach to monitor the aging behavior of luminescent materials. The results demonstrate that this synthetic approach provides a reliable route to address the instability issue of perovskite QDs, while the sonification strategy expands the functional applications of such materials in interactive systems.

## Data Availability

The data that support the findings of this study are available from the corresponding author upon reasonable request. The data are not publicly available because they are part of an ongoing study.

## Supplementary Materials

The following supporting information can be downloaded at https://www.mdpi.com/article/10.3390/nano16201290/s1, Audio S1: Sonification of Pristine CsPbBr_3_ QDs under Cyclic Heating–Cooling; Audio S2: Sonification of pristine CsPbBr_3_ QDs under temperature-dependent aging; Audio S3: Sonification of pristine CsPbBr_3_ QDs under UV aging; Audio S4: Sonification of Zn/CsPbBr_3_-TBP QDs under cyclic heating–cooling; Audio S5: Sonification of Zn/CsPbBr_3_-TBP QDs under temperature-dependent aging; Audio S6: Sonification of Zn/CsPbBr_3_-TBP QDs under UV aging; Audio S7: Overlay of All six sonified fragments for simultaneous comparison. Figure S1. (a) PL spectra and (b) UV-vis absorption spectra of CsPbBr_3_ QDs and CsPbBr_3_-TBP QDs; Figure S2. XRD spectra of CsPbBr_3_ and CsPbBr_3_-TBP QDs; Figure S3. (a) Full spectrum of CsPbBr_3_ QDs and CsPbBr_3_-TBP QDs, and high resolution XPS spectra of (b) Cs 3d, (c) Pb 4f, (d) Br 3d; Figure S4. (a,b) TEM images, and (c,d) grain size distributions of CsPbBr_3_ QDs and CsPbBr_3_-TBP QDs; Figure S5. Temperature dependent PL spectra of (a) CsPbBr_3_ QDs and (b) CsPbBr_3_-TBP QDs; Figure S6. PL spectra of (a) CsPbBr_3_ QDs and (b) CsPbBr_3_-TBP QDs after cyclic heating to 100 °C and cooling to room temperature; Figure S7. PL spectra of (a) CsPbBr_3_ QDs and (b) CsPbBr_3_-TBP QDs irradiated at 365 nm light for 10 h; Table S1. Summary of PL intensity retention rates for four QD samples under three aging conditions.
