# Supplementary material for: Musical Sonification of Ligand-Engineered CsPbBr3 Quantum Dots with Enhanced Stability
Source: Nanomaterials (Basel). 2026 Oct 11;16(20):1290. doi: 10.3390/nano16201290 (PMC13707871; doi:10.3390/nano16201290)
Supplement: Supplementary file 1 [file nanomaterials-16-01290-s001.zip › Supporting Information.pdf]

From PL Spectra to Sonic Signatures: Ligand Engineering of $CsPbBr_3$ Quantum Dots for Enhanced Stability and Interactive Display Applications

Cheng Sun [1], Yajuan Wang [2,4], Zhiyang Xu [1], Jianing Wang [3] and Wenyan Liu [2,*]

[1] Department of Music, Beihua University
[2] Department of Materials Science and Engineering
[3] Changchun Institute of Optics, Fine Mechanics and Physics, Chinese Academy of Sciences
[4] Department of Basic Disciplines, Nanchang Jiaotong University
* Correspondence: wyliu1234@163.com; Tel.: +86-18604495978

The following supporting information is provided for this manuscript. Seven audio files (Audio S1-S7) are supplied as separate electronic files alongside this document. These files were generated from the sonification of photoluminescence (PL) spectral data of $CsPbBr_3$ quantum dots (QDs) under various aging conditions, following a mapping scheme in which PL intensity is converted to pitch (higher intensity→higher pitch) and PL peak position is converted to dynamic level (larger peak shift→softer dynamics). Audio S1-S6 correspond to the six individual sonified fragments for pristine and Zn/$CsPbBr_3$-TBP QDs under cyclic heating-cooling, temperature-dependent, and UV aging conditions, respectively. Audio S7 is an overlay of all six fragments combined into a single track, enabling a simultaneous auditory comparison of the six aging trajectories.

Additional supplementary figures and tables are listed below.

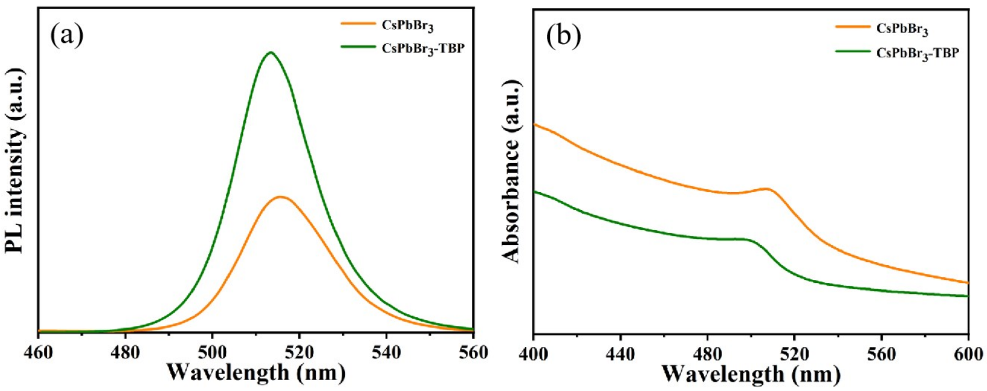

Figure S1 (a) PL spectra and (b) UV-vis absorption spectra of $CsPbBr_3$ QDs and $CsPbBr_3$-TBP QDs

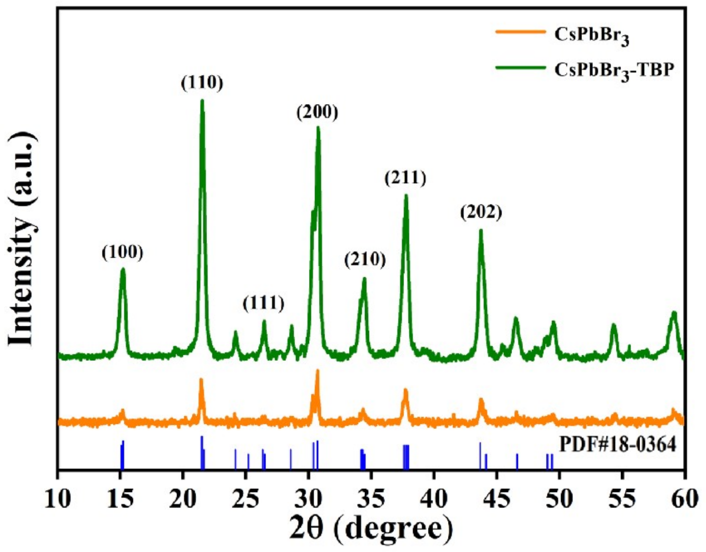

Figure S2 XRD spectra of $CsPbBr_3$ and $CsPbBr_3$-TBP QDs

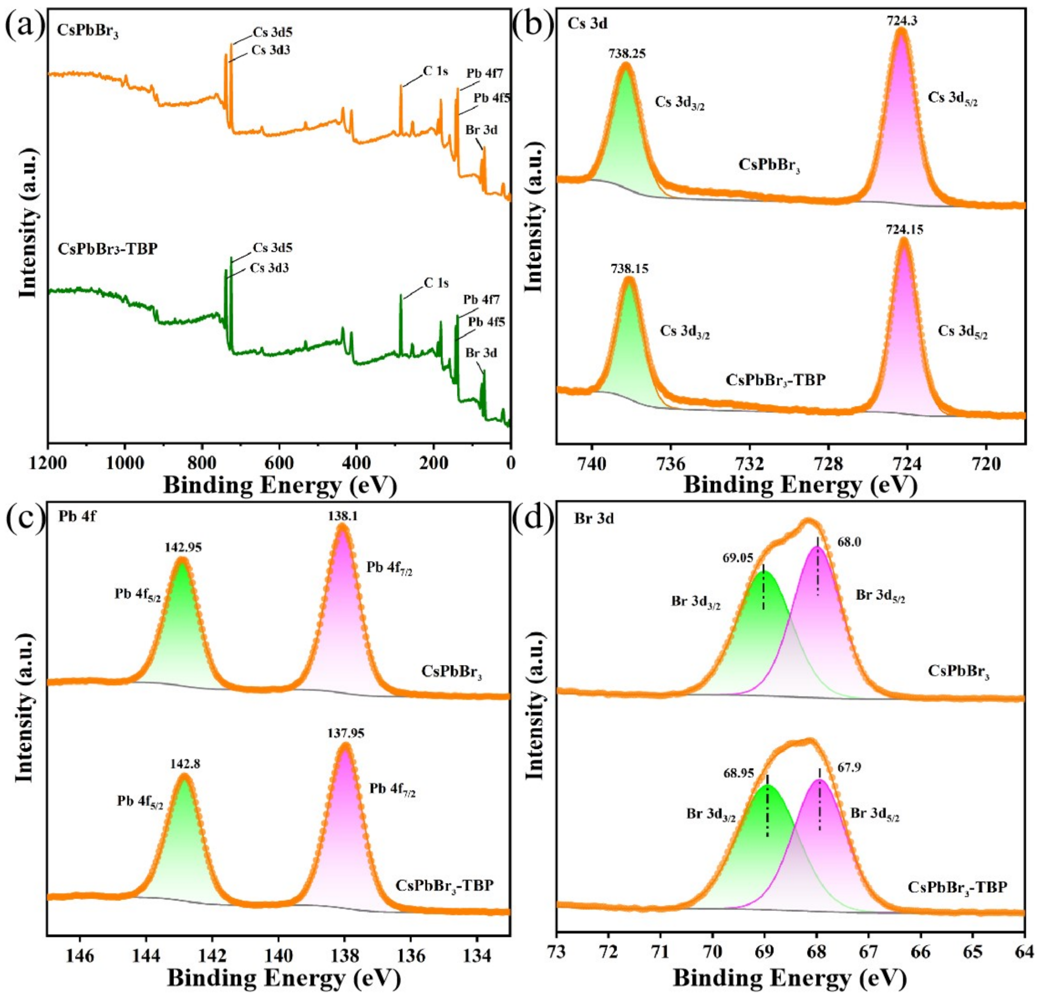

Figure S3 (a) Full spectrum of $CsPbBr_3$ QDs and $CsPbBr_3$-TBP QDs, and high resolution XPS spectra of (b) Cs 3d, (c) Pb 4f, (d) Br 3d

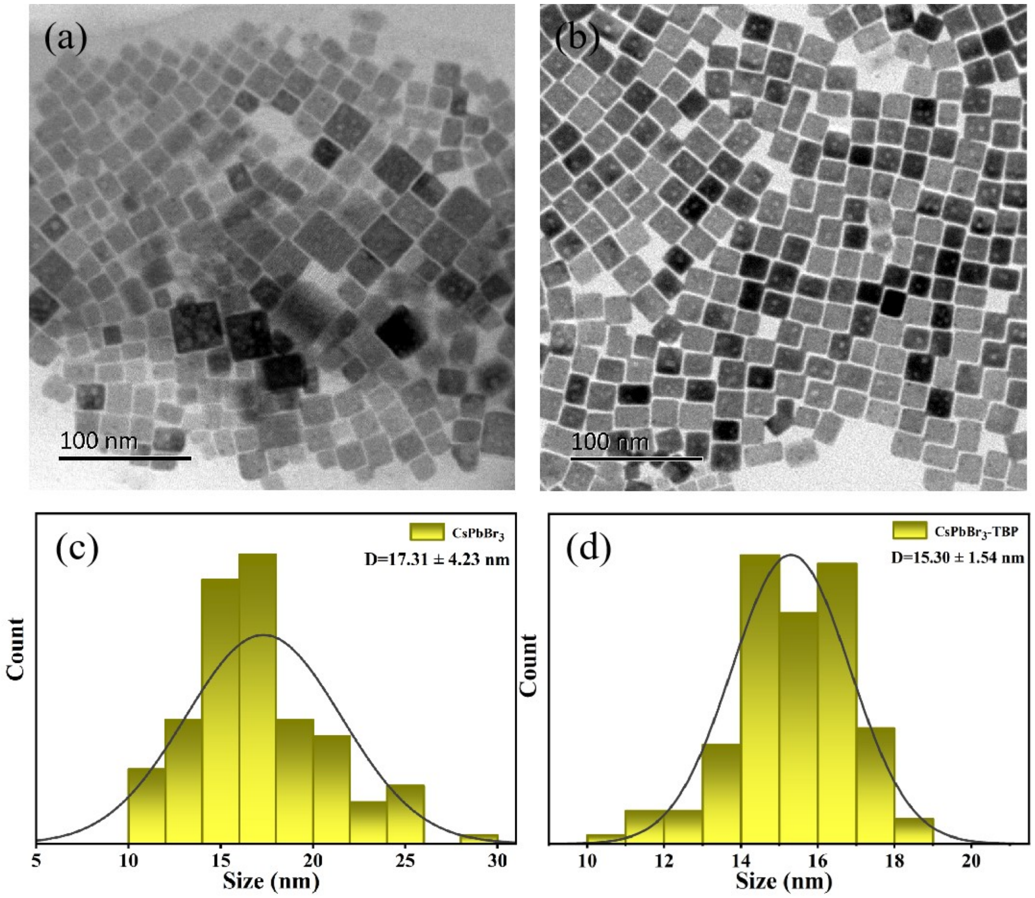

Figure S4 (a, b) TEM images, and (c, d) grain size distributions of $CsPbBr_3$ QDs and $CsPbBr_3$-TBP QDs

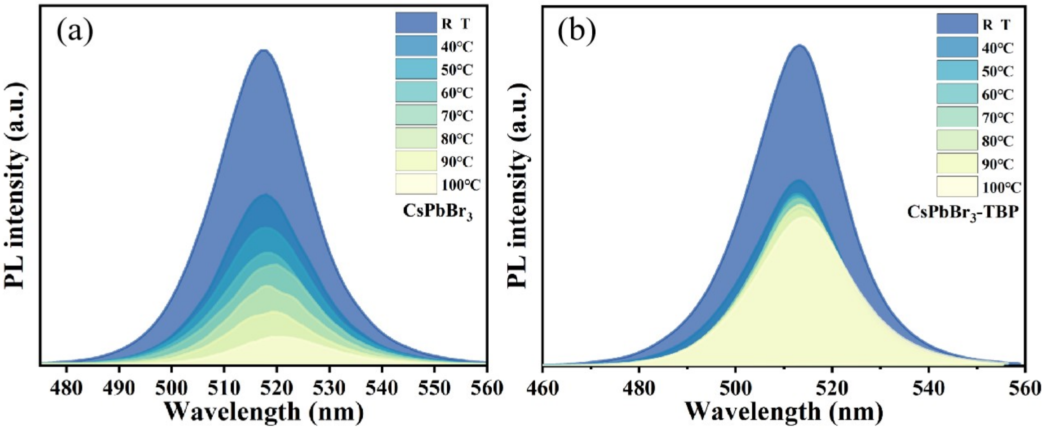

Figure S5 Temperature-dependent PL spectra of (a) $CsPbBr_3$ QDs and (b) $CsPbBr_3$-TBP QDs

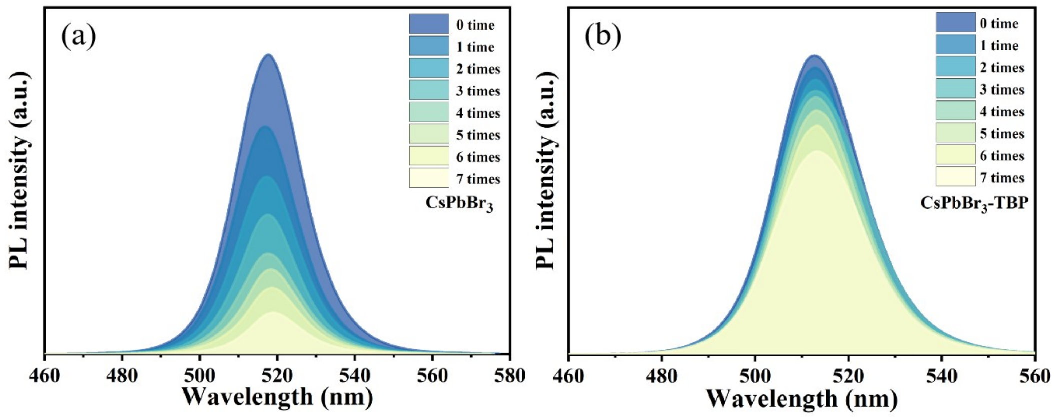

Figure S6 PL spectra of (a) $CsPbBr_3$ QDs and (b) $CsPbBr_3$-TBP QDs after cyclic heating to 100°C and cooling to room temperature

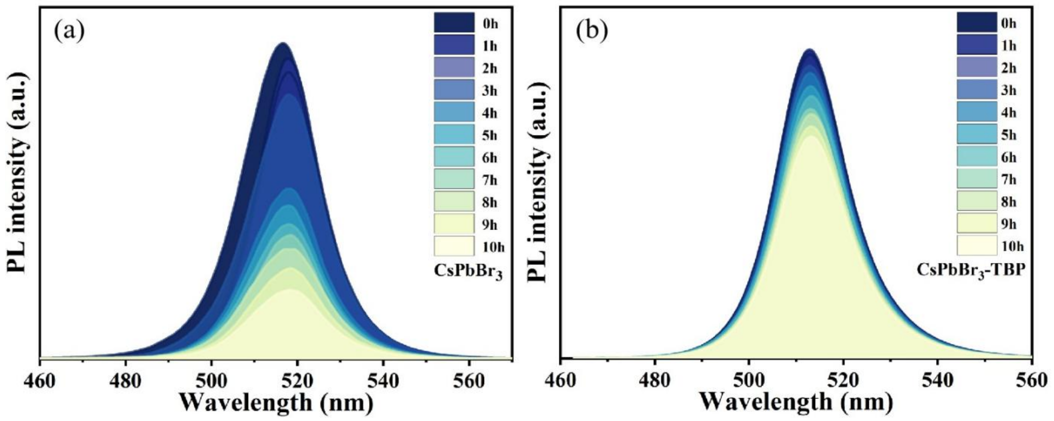

Figure S7 PL spectra of (a) $CsPbBr_3$ QDs and (b) $CsPbBr_3$-TBP QDs irradiated at 365 nm light for 10 hours

Table S1 Summary of PL intensity retention rates for four QD samples under three aging conditions

Sample	PL retention at 100 °C	PL retention after 7 heating-cooling cycles	PL retention after 10 h UV irradiation
$CsPbBr_3$	8%	13%	21%
$CsPbBr_3$-TBP	46%	67%	72%
Zn/$CsPbBr_3$	15%	41%	46%
Zn/$CsPbBr_3$-TBP	63%	79%	73%
